# Diagnostic hypotheses and acute tomographic findings in the emergency room of a tertiary hospital: an accuracy study

**DOI:** 10.1590/0100-6991e-20263891-en

**Published:** 2026-01-27

**Authors:** MEIR EDUARDA DA ROCHA DOS SANTOS, MATHEUS ALBERTO CELLA, ALISSA SCHIMIDT SAN MARTIN, KALIANDRA MENEZES CANTON, LUCIANA ESTACIA AMBROS, JORGE ROBERTO MARCANTE CARLOTTO

**Affiliations:** 1 - Universidade de Passo Fundo, Escola de Medicina - Passo Fundo - RS- Brasil

**Keywords:** Emergency, Diagnostic Hypothesis, Image Report, Accuracy, Computed Tomography, Emergência, Hipótese Diagnóstica, Laudo de Imagem, Acurácia, Tomografia Computadorizada

## Abstract

**Introduction:**

Growing technological innovation represents an advance in medical decision-making. However, the large number of computed tomography (CT) scans performed in the emergency department is worrying due to the costs generated by the hospital and the exposure of patients to ionizing radiation. The availability and performance of the exam, however, does not necessarily mean an increase in diagnoses, and some of these exams may not show any pathology or alteration. Thus, our study aims to evaluate the accuracy between the emergency physician’s diagnostic hypothesis and the results of the requested tomography of the skull, thorax and abdomen segments.

**Methodology:**

Prospective cross-sectional study between September 2023 and February 2024. The sample consisted of 331 patients, the inclusion criteria being: majority, having undergone a tomographic examination of the skull, chest or abdomen and having signed the Free and Informed Consent Form (ICF).). Statistical analysis was performed using IBM SPSS Statistics 27.0 software.

**Results:**

409 CT scans were analyzed, of the CT scans performed, 49.74% were devoid of acute findings. Proportion of agreement with the initial clinical diagnostic hypothesis was 39.5% in the skull, 40.3% in the abdomen and 43.7% in the thorax.

**Conclusion:**

This study identified low agreement between clinical hypotheses formulated in the emergency room of a tertiary hospital and acute tomographic findings, highlighting situations in which the use of CT was considered unnecessary for formulating the final diagnosis. However, it is pertinent to highlight the limitations of the study, above all, the impossibility of directly evaluating the reasons why each exam was requested within the subjective nuances of clinical practice.

## RESUMO

**Objetivo:**

A crescente inovação tecnológica representa um avanço para tomada de decisões médicas. Entretanto, o vultoso número de tomografias computadorizadas (TC) realizadas no setor de emergência é preocupante em virtude dos custos gerados ao hospital e da exposição dos pacientes à radiação ionizante. A disponibilidade e realização do exame, todavia, não significa necessariamente aumento de diagnósticos, podendo uma parcela desses exames não apresentar nenhuma patologia ou alteração. Assim, nosso estudo objetiva avaliar a acurácia entre hipótese diagnóstica do emergencista e resultado da tomografia solicitada dos segmentos crânio, tórax e abdome.

**Metodologia:**

Estudo transversal prospectivo entre setembro de 2023 e fevereiro de 2024. A amostra foi 331 pacientes, sendo os critérios de inclusão: maioridade, ter realizado exame tomográfico de crânio, tórax ou abdome e ter assinado o Termo de Consentimento Livre e Esclarecido (TCLE). A análise estatística foi realizada através do software IBM SPSS Statistics 27.0.

**Resultados:**

Foram analisadas 409 tomografias, das TCs realizadas 49,74% foram desprovidas de achados agudos. Proporção de concordância com hipótese diagnóstica clínica inicial foi 39,5% no crânio, 40,3% no abdome e 43,7% no tórax.

**Conclusão:**

Este estudo identificou baixa concordância entre hipóteses clínicas formuladas no pronto atendimento de um hospital terciário e achados agudos tomográficos, evidenciando situações nas quais o uso da TC foi considerado dispensável à formulação do diagnóstico final. Entretanto, é pertinente ressaltar as limitações do estudo, sobretudo, a impossibilidade de avaliar diretamente as razões pelas quais cada exame foi solicitado dentro das nuances subjetivas da prática clínica.

## INTRODUCTION

Best patient care is the primary objective in any health system. However, there is a complex dichotomy between imaging exams and physical examination, since there has been a great technological advance, contributing to several diagnoses, especially those that depend on time to perform surgical treatment. The number of computed tomography (CT) scans performed in emergency rooms (ERs) has risen excessively, increasing hospital costs and patient exposure to ionizing radiation. In Brazil, the number of CT scans increased from 540,067 to 921,485 between 2008 and 2011 in the Public Unified Health System (SUS)^1^, which does not necessarily mean more diagnoses, as a portion of these scans may not present any pathology or alteration.

Cranial (Skull) CT has become the modality of choice for evaluating acute neurological complaints of patients treated in ERs and its use has grown significantly in recent years, although not followed by a proportional increase in pathologies diagnoses^2^. One contributing factor to this rise is the challenge physicians face in determining which patients require further investigation to clarify the source of their symptoms and subsequent treatment, as opposed to those who can be discharged without additional testing to inform medical decisions^3^. This highlights the difficulty in establishing diagnosis only with clinical history and physical examination. Considering this, the accuracy is based on the extent to which the diagnostic hypothesis formulated in the ER agreed, mainly through medical semiology, with the findings present in the imaging studies. 

As for chest CT, the reasons that justify its request are related in most cases to cardiac and pulmonary pathologies, in addition to blunt thoracic trauma. In the case of the latter, CT has been very effective in detecting lesions, therefore resulting in faster patient management. Nonetheless, findings upon physical examination, such as pain at palpation, also demonstrate a relationship with the presence of lesions in the chest^4^. Therefore, the initial clinical examination is essential for formulating diagnostic hypotheses and determining appropriate medical management and should not be substituted by tomography.

According to the study by Silva et al (2019), which evaluated the diagnoses of abdominal diseases in the ER of a private hospital of high complexity pre-CT, post-CT, and final diagnosis, abdominal CT scans correctly showed diseases’ findings in 92-97% of patients, presented normal results in 44% of patients, and corresponded with clinical data in 70% of cases. Among the most frequent diagnoses presented in the study were nonspecific abdominal pain, obstructive uropathy, and appendicitis, which together corresponded to 73.6% of all clinical diagnoses, 58.5% of post-CT diagnoses, and 61.3% of definitive diagnoses^1^.

Most studies so far have assessed the prevalence of tomographic findings or data on imaging findings in hospital ERs, lacking information about the accuracy between the clinical diagnosis and its agreement with the imaging tests requested after initial care. Therefore, the main objective of the present study is to evaluate the accuracy between the diagnostic hypothesis formulated by the ER physician and the acute tomographic findings of the cranial, thorax, and abdominal segments of patients treated in a SUS tertiary hospital.

## METHODS

This was a cross-sectional study conducted at Hospital São Vicente de Paulo (HSVP), in Passo Fundo, State of Rio Grande do Sul (RS), Brazil, with prospective data collection from September 2023 to February 2024.

### Population and sample

We included 331 patients treated at the emergency department of HSVP who underwent Computed Tomography of the Abdomen, Chest, or Skull, with or without contrast, to evaluate acute findings. Some of the included patients underwent CT scans of two or three segments, totaling 409 analyzed exams. Each CT scan was assessed separately and counted according to its respective anatomical segment.

### Inclusion Criteria

Individuals over 18 years of age who were admitted to the HSVP ER through SUS and underwent Computed Tomography of Thorax, Abdomen, or Skull with or without contrast were included for the evaluation of acute findings (described in Tables 1, 2, and 3).

**Table 1:** Correlation between clinical hypotheses and tomographic findings in the cranial segment.

Diseases	Clinical Hypotheses	Radiological findings
Stroke	70	19
Non-traumatic intracranial hemorrhage	9	9
Non-traumatic Subarachnoid Hemorrhage	6	2
Traumatic brain injury	55	16
Traumatic Intracranial Hemorrhage	0	8
Traumatic Subarachnoid Hemorrhage	0	4
Cerebral contusion	0	2
Bone Fracture	0	2
Cerebral Edema	5	2
Hydrocephalus	11	3
Midline Deviation	9	1
Brain Mass	12	2

Source: authors.

**Table 2:** Correlation between clinical hypotheses and CT findings in the chest segment.

Diseases	Clinical Hypotheses	Radiological findings
Pneumonia	29	22
Pleural Effusion	13	12
Acute Coronary Syndrome (ACS)	3	0
Aortic Dissection	3	1
Pulmonary Embolism	2	0
Pneumothorax	3	0
Bone Fracture	26	0
Aortic Aneurysm	1	0
Pulmonary congestion	5	2
Hemothorax	2	0
Pulmonary contusion	8	5

Source: authors.

**Table 3:** Correlation between clinical hypotheses and CT findings in the abdominal segment.

Diseases	Clinical Hypotheses	Radiological findings
Aortic Dissection	2	1
Appendicitis	10	5
Visceral Perforation	37	4
Cholecystitis	9	8
Intestinal Obstruction	13	5
Pancreatitis	6	3
Mesenteric Ischemia	1	0
Abdominal Aortic Aneurysm	2	1
Diverticulitis	7	7
Enterocolitis	6	8
Obstructive Uropathy/Ureterolithiasis	15	17
Pyelonephritis	8	7
Renal Lithiasis	9	5
Pneumoperitoneum	2	0
Acute Renal Failure	2	0
Urinary Tract Infections	8	2
Subocclusion	3	6

Source: authors.

### Exclusion Criteria

Patients under 18 years of age, unconscious, hemodynamically unstable, with previously diagnosed chronic diseases or neoplasms, and exams with poor technical quality that compromised the adequate analysis.

### Variables under Study

The patients selected for the study were evaluated in terms of symptoms presented at the time of admission to the emergency department, data recorded in their medical records, with diagnostic hypotheses recorded with their respective International Classification of Diseases (ICD) code, and in terms of acute radiological findings found in computed tomography scans. Acute findings in the cranial segment were considered ischemic stroke (IS), traumatic brain injury (TBI), acute intracranial hemorrhage, and cerebral mass - acute cerebral edema. 

The acute chest radiological findings considered were acute pulmonary embolism, pneumothorax, hemothorax, acute pleural effusion, pulmonary contusion, and acute aortic dissection. 

The acute findings considered from the abdomen were acute intra- or retroperitoneal hemorrhage, rupture of solid viscus, acute mesenteric ischemia, acute appendicitis, acute cholecystitis, obstructive uropathy, diverticulitis, and pyelonephritis. 

Normal CT scans and chronic radiological findings that did not correlate with the patient’s acute complaint in the emergency room were considered non-acute findings. 

### Ethical Aspects

The project was approved by the Ethics in Research Committee of the São Vicente de Paulo Hospital in Passo Fundo. Acceptance and signing of the Informed Consent Form (ICF) was a mandatory criterion for the participation of all patients in the study. In addition, total confidentiality was ensured about the information collected.

### Statistical analysis

We entered the clinical and radiological data obtained into the software Statistical Package for the Social Sciences (SPSS), version 24.0, with the commercially accessible Propensity Score Matching (PSM) extension, to enable the statistical analysis and its relevance. The proportion of agreement between the diagnostic hypotheses and the clinical findings was calculated by adding the agreements by anatomical segment (cranial, thorax, and abdomen). The evaluators of the agreement between hypotheses and tomographic findings were blinded to the results of the CT scans of the respective patients.

## RESULTS

We analyzed CT scans in 331 patients admitted to the ER of a tertiary hospital in the city of Passo Fundo, Brazil. The sample consisted of 174 women (52.6%) and 157 men (47.4%), with a mean age of 53.5 years (± 5 years) for both sexes. 

Among the included patients, 263 (79.4%) underwent only one CT scan (Thorax, Abdomen or Skull), 35 (10.6%) underwent two (Thorax and Abdomen, Thorax and Skull, or Abdomen and Skull), and 33 (10%) underwent all three exams. Each scan was analyzed separately and counted according to its respective anatomical segment, even in those cases in which three exams were performed in the same patient.

Of the total number of scans performed, 99 were of the thorax (29.9%), 150 of the abdomen (45.3%), and 183 of the cranial segment (55.3%), totaling 432. There were 23 losses due to inadequate image quality, so we included 409 CT scans in the study.

We observed that 185 patients (55.9%) underwent contrast-enhanced scans, and 146 (44.1%), non-contrast-enhanced ones. Traumatic emergencies accounted for 107 (32.3%) of the patients, while non-traumatic emergencies afflicted 224 individuals (67.7%). 

In the ER care, diagnostic hypotheses were formulated to be confirmed by CT scans. Considering the hypotheses related to pathologies of the cranial segment, the three most prevalent were ischemic stroke (38.2%), traumatic brain injury (35.5%), and cerebral mass (6.5%). 

Among the hypotheses related to thoracic pathologies, the four most prevalent were pneumonia (30.2%), bone fracture (27.1%), pleural effusion (13.5%), and pulmonary contusion (8.3%). Among the hypotheses related to abdominal pathologies, the three most common were visceral perforation (26.4%), obstructive uropathy/ureterolithiasis (17.1%), and pyelonephritis (7.8%)

Brain CT scans were the ones with the least acute findings, representing 59.3% of the total, followed by chest CT (45.8%) and abdominal (42.8%).

The CT scans of the cranial segment displayed agreement between the diagnostic hypotheses and the acute clinical findings of 39.5%, i.e., the diagnostic hypothesis was confirmed in 39.5% of the CT findings. Chest CT scans, on the other hand, had agreement of 43.7%. 

Abdominal CT scans findings agreed with diagnostic hypotheses and acute clinical findings in 40.3% of cases. The diagnostic hypothesis that showed the greatest agreement with the acute tomographic findings was obstructive uropathies (9.8%). 

Stroke was the diagnostic hypothesis that most agreed with the acute tomographic findings (9.8%) in the cranial segment, whereas in the chest segment, pneumonia was the hypothesis whose acute tomographic findings obtained the greatest agreement (20.7%). 

The total number of CT scans without acute findings was 49.74%. Considering the body segments alone, exams without findings represented 59.4% of cranial scans, 47.4% of chest ones, and 43.2% of abdominal CT exams. 

## DISCUSSION

This study identified a low agreement between the clinical hypotheses formulated in the emergency room of a tertiary hospital and the acute tomographic findings, unveiling multiple situations in which the use of Computed Tomography was considered dispensable for the formulation of the final diagnosis. This fact is corroborated by the high number of unaltered CT scans found during the study, especially in the cranial segment, where most of the requested scans did not present acute findings relevant to the clinical outcome (58.4%), a premise that had already been established in a previous article similar to our study^5^ ^-^ ^6^. 

It is worth emphasizing, however, the influence that hypotheses such as intracerebral hemorrhages, subarachnoid hemorrhages, and other cerebrovascular accidents exert on the low accuracy of CT in this segment, since both computed tomography and magnetic resonance imaging are considered options of first choice for imaging tests to confirm the diagnosis in patients suspected of presenting these conditions in the emergency department^7^. That said, it can be understood that the approach to requesting imaging studies in the sector has been adequate, despite the suspicion and construction of clinical hypotheses having shown low agreement. 

A different result was observed in the chest segment, with the highest level of agreement between the clinical hypotheses and their respective imaging findings. In this group, the hypothesis whose acute CT findings were most consistent was pneumonia, for which 31.5% of the CT scans were requested for diagnostic purposes. This approach, although frequent in highly complex services, is not recommended by the current literature, there being no direct evidence to suggest a better clinical outcome in patients undergoing CT in the initial investigation^8^. 

Regarding the abdominal segment, a significant part of the CT scans requested did not show acute findings. Silva et al, in 2019, observed a small percentage (12.9%) of CT scans without findings. Despite this disagreement with our results, it is possible to observe unnecessary requests for imaging studies present in tertiary hospitals in different regions of the country^1^. 

Still in the abdominal segment, we observed the prevalence of clinical conditions related to the digestive system to the detriment of pathologies resulting from trauma or from the urinary tract. This data is in line with previous studies showing that gastrointestinal tract pathologies end up being more prevalent in several centers^9^. 

In conclusion, 49.74% of CT scans performed on Emergency Room patients of a Public Unified Health System tertiary hospital were considered unnecessary, indicating the need to implement protocols to optimize CT requests. However, it is pertinent to highlight the limitations of the study, especially since it is not possible to directly assess the reasons why each test was requested, especially within the subjective nuances of clinical practice.

As already pointed out, and despite the multifactorial etiology of the problem, the injudicious use of advanced imaging tests in emergency care services is unquestionable, a scenario that has global repercussions on health care, whether in unnecessary exposure to ionizing radiation for patients or in the increase in health spending within the national sphere^10^.

## FINAL CONSIDERATIONS

A considerable number of computed tomography scans of the cranial, thoracic, and abdominal segments requested in the Emergency Department did not present findings that would justify their requests, since a considerable part of the exams did not show pathological alterations. Thus, it is essential to question and analyze the unnecessary costs to the Unified Health System for performing CT scans in tertiary hospitals, since in the present study, in more than half of the CT scans requested, there was no agreement between diagnostic hypotheses and acute imaging findings. This highlights the need to reevaluate the diagnostic elaboration process (either by focusing on the clinical history or physical examination) and the respective request for CT scans in patients who seek care in the emergency sectors through the Unified Health System in Brazil, evidencing the need for the development and implementation of protocols and/or clinical guidelines to optimize the request for CT scans in emergency sectors.
